# Symmetrical acrokeratoderma: a case series in Indian patients

**DOI:** 10.1186/s13023-016-0541-9

**Published:** 2016-11-22

**Authors:** Keshavamurthy Vinay, Gitesh U. Sawatkar, Uma N. Saikia, Sunil Dogra

**Affiliations:** 1 Department of Dermatology, Venereology and Leprology, Postgraduate Institute of Medical Education and Research, Sector 12, Chandigarh, 160012 India; 2 Department of Histopathology, Postgraduate Institute of Medical Education and Research, Chandigarh, India

**Keywords:** Acitretin, India, Symmetrical acral keratoderma, Symmetrical acrokeratoderma

## Abstract

**Abstract:**

Symmetrical acrokeratoderma is a recently described dermatosis in young adult males of Chinese descent. In this report, we describe a series of five cases of symmetrical acrokeratoderma from India. All 5 patients had asymptomatic, brownish-black plaques distributed symmetrically over dorsum of hand and feet with variable involvement of wrist, flexural surface of forearm, elbow, ankles, shin and knee joint. Palms and soles were characteristically spared. Typically whitish maceration of the lesions was seen after immersing in water. The disease showed exacerbation in hot and humid climate with spontaneous resolution in winter. Histopathological examination showed basket weave hyperkeratosis, irregular acanthosis and mild peri-vascular lymphomononuclear infiltrate. Loosening of the stratum corneum was seen in post-immersion biopsy specimens. Acitretin appeared to provide symptomatic improvement in the short term without any long-term effect on the natural disease course. The genetic and environmental factors involved in disease causation needs to be elucidated in future.

**Trial registration:**

The study was not registered in a trial registry since it was a retrospective analysis of the clinical records and not an interventional/observational study.

To the Editor,

Symmetrical acrokeratoderma is a recently described dermatosis with distinct clinical features [1–3]. All published cases till date has been reported in patients of Chinese descent. Herein we report a series of five patients of symmetrical acrokeratoderma from India and describe the natural history, clinical, epidemiological and histopathological features and treatment outcome.

A total of five patients who fulfilled the previously proposed diagnostic criteria for symmetrical acrokeratoderma were identified from our records [2]. Briefly, a diagnosis of symmetrical acrokeratoderma was established in patients who had (1) brown to black symmetrically distributed hyperkeratotic patches over acral sites but sparing palms and soles; and (2) the lesions became white and macerated rapidly after water immersion or sweating, but recovered gradually after drying [2]. All patients were males with age range of 11-31 years, belonged to the Punjab province of India and presented to us in the summer months of May, June and July 2015. Four patients had their disease onset during adolescence with mean disease duration of 6.25years (4-8 years). One boy manifested his symptoms since infancy and presented to us at the age of 11 years. The demographic and clinical features are tabulated in Table 1.

**Table 1:** Clinical and epidemiological features and treatment response of symmetrical acrokeratoderma

Patient No	Age at onset (Years)/Sex	Total disease duration (Years)	Sites involved	Family history	Seasonal variation and natural course	Associated cutaneous diseases	Follow-up duration (months)	Treatment offered/duration of treatment	Response to treatment
1	24/M	6	Dorsum of hand and feet, wrist, flexural surface of forearm, ankle, shins, knee joint	No	Summer exacerbation. Resolves completely in winter	No	14	Topical retinoic acid 0.1% gel and 10% urea	Mild improvement. Relapse in summer
2	11/M	11	Knuckles, interphalangeal joint, wrist, elbow	No	Summer exacerbation. Resolves completely in winter	No	14	Topical retinoic acid 0.1% gel + 10% urea	No improvement
								Acitretin 10 mg/day* 16 weeks. Topical 10% urea	Cleared completely
3	21/M	7	Knuckles, wrist, ankle	No	Summer exacerbation. Mild improvement in winter	No	15	Acitretin 25mg/day* 12 weeks. Topical 10% urea	Cleared completely. Seasonal recurrence.
4	28/M	8	Dorsum of hand and feet Forearm	No	Summer exacerbation. Resolves completely in winter	No	15	Acitretin 25mg* 4 weeks. Topical 10% urea	Cleared completely. Seasonal recurrence.
5	31/M	4	Dorsum of hand and feet, forearm and shin	No	Summer exacerbation. Resolves completely in winter	Palmoplantar hyperhydrosis	12	Acitretin 25 mg* 8 weeks 10mg* 16 weeks. Topical 10% urea	Cleared completely. Seasonal recurrence.

*M* male

Uniformly, the patients complained of insidious onset, asymptomatic, brownish black plaques (often referred as dirt) distributed over dorsum of hand and feet with variable involvement of wrist, flexural surface of forearm, elbow, ankles, shin and knee joint (Fig. 1a). Palms and soles were characteristically spared. Typically all patients had noticed whitish maceration of the lesions after immersing in water or sweating and spontaneous recovery on drying. The same could be elicited clinically by immersing the patient’s hands and feet in water for 5 minutes (Fig. 1b). Patients consistently complained of exacerbation of symptoms in hot and humid climate during summer months and in four of the patients, the disease showed spontaneous resolution in winter. Atopic diathesis and icthyosis vulgaris was not seen in any of the patients and only one patient suffered from palmoplantar hyperhidrosis.

**Fig. 1:**
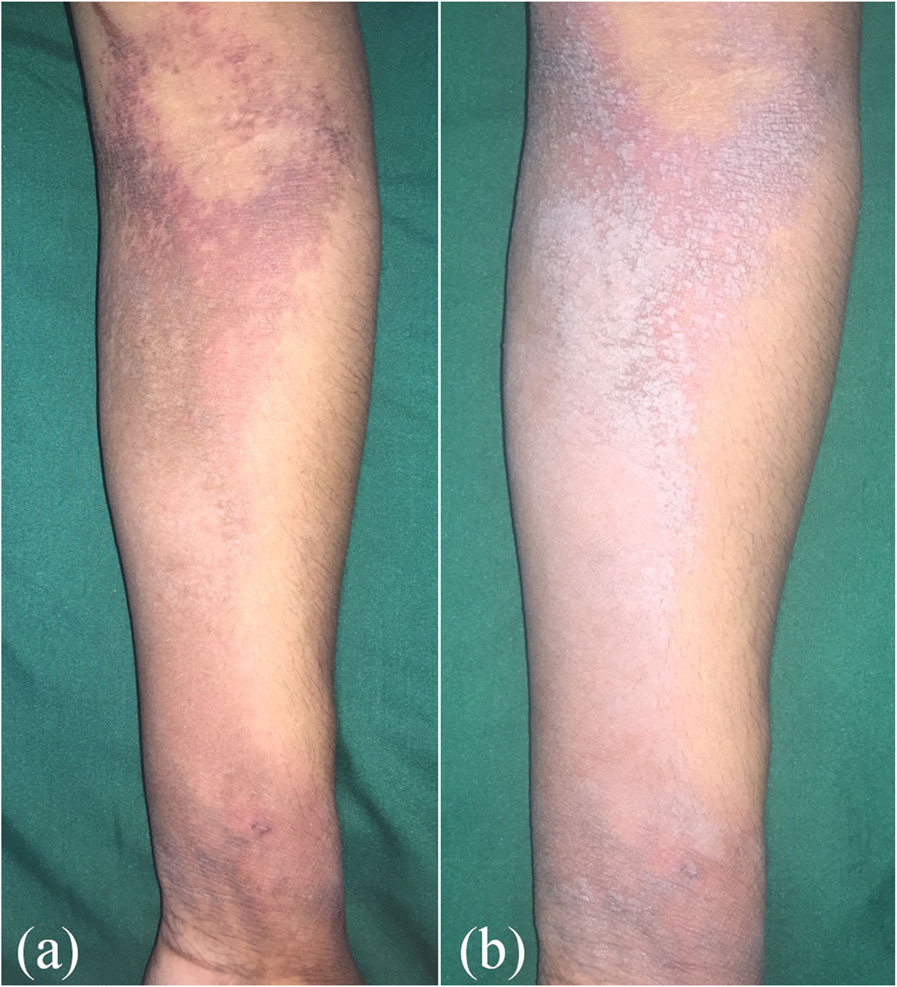
**a** Case of symmetrical acrokeratoderma showing brownish black plaque over flexural aspect of left forearm. **b** White maceration after immersion in water for five minutes

Histopathological examination consistently showed basket weave hyperkeratosis, normal granular layer and irregular acanthosis (Fig. 2a). Increased pigmentation at papillary tips was noted in three patients. Histopathological analysis of the post-immersion specimen showed loosening of the stratum corneum (Fig. 2b). No fungal elements were seen in any of the biopsy specimens.

**Fig. 2:**
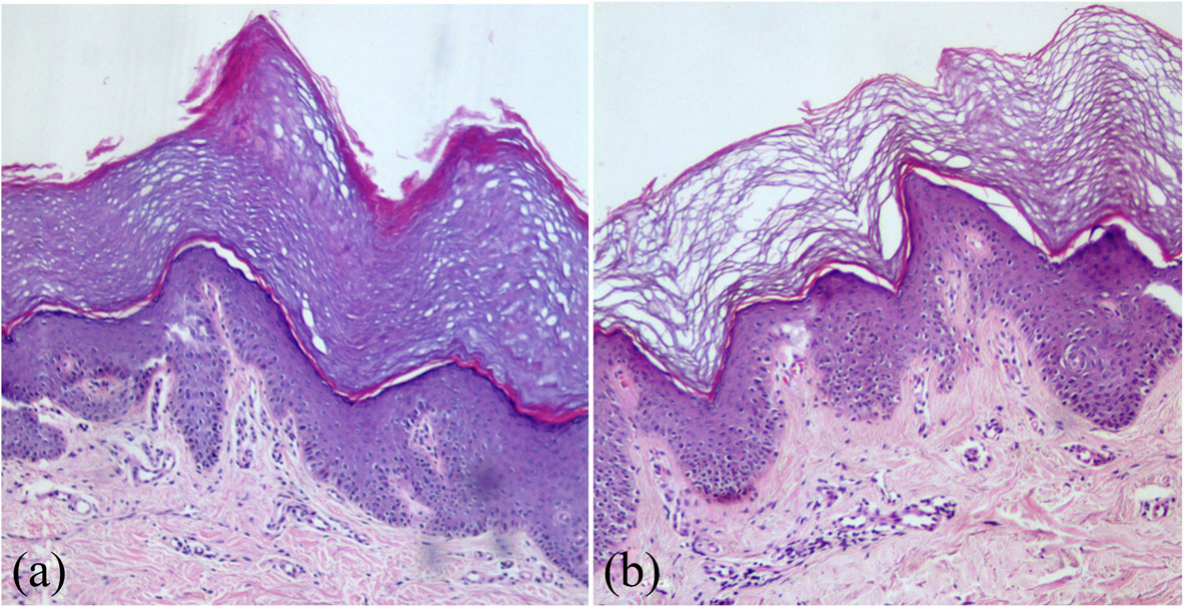
**a** Microphotograph of symmetrical acrokeratoderma showing basket weave orthokeratosis, irregular acanthosis and mild perivascular lymphocytic infiltrate (H&E, 100X). **b** Microphotograph of post-immersion skin biopsy showing loosening of the stratum corneum (H&E, 100X)

Most of the patients had been diagnosed in the past as acral acanthosis nigricans, Addisonian pigmentation, psoriasis, frictional keratosis and aquagenic syringeal acrokeratoderma (ASA) and had received emollients, topical corticosteroids and topical salicylic acid with minimal benefit. Oral acitretin with or without topical retinoic acid 0.1% gel and 10% urea in glycerine was used to treat these cases (Table 1). There was considerable improvement in skin texture and maceration within 4 weeks of starting treatment. All patients responded to treatment with complete clearance of lesions. Oral acitretin was gradually tapered and stopped. However, in three patients, the disease relapsed with the onset of summer. These three patients were re-started on acitretin to which they responded favorably.

Symmetrical acrokeratoderma is a rare dermatosis with only a handful of cases reported in the literature. Fan et al. [1] in 2010 were the first to report symmetrical acrokeratoderma in the English literature. The authors also reviewed the previously published cases in the Chinese literature. Subsequently, Liu et al. [2] and Li et al. [3] have reported their experience of symmetrical acrokeratoderma in 34 and 71 patients respectively. Interestingly, all the previously reported cases were in patients of Chinese descent. To the best our knowledge, ours is the first series reporting symmetrical acrokeratoderma outside of China.

Our patients shared similar clinical and epidemiological features as reported previously. The disorder predominantly affected young men, had minimal subjective symptoms, involved acral parts with sparing of palms and soles, had summer exacerbation/recurrence and alleviation in winter, and histopathological features of epidermal acanthosis and hyperkeratosis [1–3]. Interestingly one of our patients had skin lesions since infancy. The child was apparently normal at birth, but developed brownish-black discoloration and white maceration of acral skin by the age of 20 days. The patient had an elder sibling who was clear of any cutaneous lesions. The earliest age of onset for this condition is reported as 2 years [2]. Such an early age of onset as in an infant in our study may suggest the role of endogenous factors in disease pathogenesis. Therefore, as argued by Liu et al. [2] we prefer the term “symmetric acrokeratoderma” over the term “acquired symmetric acrokeratoderma” as suggested by Fan et al. [1].

Previous reports have identified association between symmetric acrokeratoderma and atopy, icthyosis vulgaris and hyperhidrosis [2, 3]. However, no such association was noted in our series. Earlier reports in Chinese literature implicated Malassezia as a causative factor. However, most of the recent reports agree on Malassezia being a “bystander” commensal [1–3]. In line with this, no fungal elements were seen on histopathological examination in our series.

The unique feature that differentiates symmetrical acrokeratoderma from other acquired/inherited keratoderma is the white maceration seen after exposure to water. However, a close clinical differential is ASA [4]. Unlike our cases, ASA patients suffer from burning sensation and palmar erythema after water immersion. Palms and soles are predominantly affected in ASA, whereas these are spared in symmetrical acrokeratoderma [4].

Trans-epidermal water loss is reported to be high and skin hydration values are low in patients with symmetrical acrokeratoderma [3]. Ultrastructural studies have shown tight clumps or aggregates of keratin tonofilaments in the perinuclear cytoplasm [5]. Partial splitting of the desmosomes in the stratum spinosum has also been reported [1] but desmosomes were found to be normal in subsequent study [5]. Therefore abnormality in the epidermal barrier function may be responsible for the unique changes observed after water immersion. On light microscopy, we could appreciate loosening of the stratum corneum after water immersion but rest of the epidermis was apparently normal.

In conclusion, symmetrical acrokeratoderma is a distinct disorder predominantly affecting young men of Asian descent. Acitretin appears to provide symptomatic improvement in the short term without any effect on the natural disease course. The genetic and environmental factors involved in disease causation needs to be elucidated in future.
